# Factors influencing care-seeking behaviour for mental illness in India: a situational analysis in Tamil Nadu

**DOI:** 10.1093/pubmed/fdab131

**Published:** 2021-10-08

**Authors:** Anant Jani, Sindhu Ravishankar, Naresh Kumar, J Vimitha, Soleil Shah, Anees Pari, C Ramasubramaniam

**Affiliations:** Oxford Martin School, University of Oxford, Oxford, OX1 3BD, UK; Oxford India Centre for Sustainable Development, Somerville College, University of Oxford, Oxford, OX2 6HD, UK; Oxford Department of International Development, University of Oxford, Oxford, OX1 3TB, UK; JSS Institute of Naturopathy and Yogic Sciences, Coimbatore, Tamil Nadu 641105, India; South India Aids Action programme, Chennai, Tamil Nadu, 600041, India; Stanford University Medical School, Stanford, CA, 94305, USA; East of England and Midlands, Public Health England, Nottingham, NG2 4UU, UK; Tamil Nadu Ministry of Health, Chennai, Tamil Nadu, 600 009, India

**Keywords:** health services, management and policy, mental health

## Abstract

**Background:**

The contribution of mental illness to the total burden of disease in India nearly doubled from 1990 to 2017, increasing from 2.5% of the total disability-adjusted life years in 1990 to 4.7% in 2017. Despite efforts by the Indian government, a treatment gap of 75–85%, with heterogeneity across multiple dimensions, exists across India. We conducted a qualitative study in Tamil Nadu, India, to better understand the contextual factors affecting the care-seeking behaviour for mental illness.

**Methods:**

Qualitative methods, including semi-structured interviews and focus groups (FGs), were conducted with stakeholders involved in the mental health care pathway in Tamil Nadu. Ten semi-structured interviews and five FGs were conducted and analysed using an inductive approach to identify codes, using Dedoose v7, related to the emerging themes and categories.

**Results:**

Our analyses identified three key areas that influence care-seeking: views on what causes and/or constitutes mental illness, stigma and discrimination associated with mental illness and broader factors influencing decision-making.

**Conclusions:**

The specific contextual factors identified by our study can be used to design and implement approaches that can help to address some of the issues that influence the care-seeking behaviour and manifest in the treatment gaps seen in Tamil Nadu and in India, more generally.

## Background

The contribution of mental illness to the total burden of disease in India nearly doubled from 1990 to 2017, increasing from 2.5% of the total disability-adjusted life years (DALYs) in 1990 to 4.7% in 2017.^1^ In 2017, it was estimated that nearly 15% of people in India were affected by mental illness with worrying increases in adults and significant variation between different states.^1^

Relatively early in its independence, India recognized the need to address the mental health needs of its citizens. India was the first postcolonial ‘non-white’ independent country to have mental health reforms. The national mental health programme (NMHP), created in 1982, established an integrated approach to mental health care delivery, utilizing a specialist and non-specialist workforce. In 1996, the NMHP was relaunched as the District Mental Health Programme which mandated the creation of mental health units at a district level. There were some serious shortcomings identified for the NMHP particularly because India ratified the United Nations Charter on Disabilities Rights and, in 2017, the Indian government introduced a rights-based Mental Healthcare Act, which replaced the Mental Healthcare Act of 1987.^1^^,^^2^

Despite the efforts by the Indian government to improve the structural elements of mental health services, policy implementation has been poor and heterogeneous across the country. In addition to the structural elements, there is also a well-documented lack of mental health resources (including staff and medications) on the ground, poor implementation of mental health services and poor adherence to evidence-based treatment, all of which contribute to the variation in mental health care-seeking behaviour, access and outcomes.^3^ In aggregate, these factors manifest themselves as an approximately 75–85% treatment gap that is heterogeneous across multiple dimensions.^3^ For example, we see differences in the treatment delivered to individuals of different ages and genders as well as in rural versus urban areas and to individuals with different severities of the illness.^3–5^

Studies aimed at understanding care-seeking behaviour have identified some important factors of mental health care pathways, but these studies often focus on the perspectives of one or two stakeholders, usually patients and carers.^6–19^ In this manuscript, we explore the mental health care pathways in Tamil Nadu, a state in Southern India, from a multi-stakeholder perspective (patients, health care professionals, non-governmental organizations (NGOs) delivering mental health services and policymakers) to identify the factors that influence the care-seeking behaviour for mental illness in India.

## Methods

Qualitative methods including semi-structured interviews and focus groups (FGs) were conducted with stakeholders who were involved in the mental health care pathway in Tamil Nadu, including government officials/policymakers, health care staff and clients (i.e. those with lived experience of mental illness and who received services for their mental illness from the NGO) and staff of NGOs involved in mental health care provision, such as providing emergency care shelter for homeless persons with psychosocial disabilities, well-being outpatient clinics and community-based mental health care. Inclusion and exclusion criteria for participants are highlighted in Table 1.

**Table 1:** Inclusion and exclusion criteria and recruitment methods of interview and FG participants

*Participant*	*Inclusion criteria*	*Exclusion criteria*	*Recruitment*
Government official, policymaker	Any professional involved with the development or execution of mental health policy either at the state or national level	N/A	Pre-identified key health care workers and policymakers with relevant experience of mental health delivery were contacted by phone or email to schedule individual interviews
Health care staff	-Doctors, nurses, counsellors, community health workers, NGO staff who work directly with individuals suffering from mental illness -Part of the public sector	-Anyone under the age of 18 -Those who are part of only the private sector (individuals who are part of both the private and public sectors can be included) -Those who only practice traditional forms of medicine such as Ayurveda or Unani	
Clients/staff of NGOs	-Over the age of 18 -Have the cognitive ability and autonomy to choose to participate and participate in the study	-Individuals with compromised autonomy -Mental health patients who are not willingly part of a NGO/self-help group -Those diagnosed with severe psychiatric disorders that affect day-to-day cognition or autonomy. These may include: -Classified by ICD as organic, including symptomatic, mental disorders (those with aetiology in cerebral disease, brain injury) -Classified by ICD as disorders due to psychoactive substance use -Classified by ICD as mood (effective) disorders ‘with’ psychotic symptoms -Classified by ICD as dissociative or somatic disorders (under neurotic, stress-related and somatoform diseases)	Pre-identified NGOs involved in mental health care provision were sent an introductory email with information on the study. We collaborated with interested NGOs to get ethics approval through their review boards. Once ethics approval was obtained, staff and NGO clients were recruited to participate on a voluntary basis. Through the ethics review, we collaborated with the NGOs to determine contextually appropriate and ethically sensitive methods to recruit NGO clients with lived experience of mental illness (i.e. distributing informational fliers, speaking with clients before/after self-help group and email list serve). NGOs were primarily responsible for recruitment and personal details of clients were not revealed to interviewers. In cases that we were unable to acquire ethical approval from the NGO due to time constraints, only NGO staff were interviewed

Two researchers trained in conducting qualitative interviews and FGs were involved in data collection. All interviews and FGs were conducted in the local language, Tamil. The data collection process lasted from March to October 2015. Ethical approval was received from the University of Oxford’s Social Sciences and Humanities Inter-divisional Research Ethics Committee and the Institutional Ethics Committee of the Indian institute of Public Health, Gandhinagar, India. The semi-structured interviews and FGs were conducted based on Interview and Focus Group guides in Supplementary Material 1.

Participants for the semi-structured interviews consisted of policymakers and mental health care professionals, while the FGs included NGO staff members and NGO clients. Participant recruitment methods are described in Table 1 and, once recruited, all participants were given a verbal explanation and text-based description of the study and its purpose in their local language, Tamil. All participants gave written consent to participate in the study after going through the information.

All interviews and FGs were recorded verbatim with a digital voice recorder, transcribed into Tamil, then translated into English, put into a common format and cross-checked by the research team. The interviews and FGs were analysed by three different researchers using an inductive approach.^20^ Briefly, analysis involved close reading of the text by three different researchers, with each researcher independently identifying the codes related to the emerging themes and categories. Codes were finalized based on a consensus between the three researchers. Once the codes were finalized, Dedoose (version 7) was used to facilitate coding of the interview and FG outputs and aggregating the relevant text under the different codes. Coding of the text enabled the analysis of emerging themes from the interview and FG text within and across different stakeholder groups.^20^ This led to the identification of three major themes agreed by the three researchers (views on what causes and/or constitutes mental illness, stigma and discrimination associated with mental illness and broader factors influencing decision-making), which served as the framework for our situational analysis.

## Results

Ten semi-structured interviews and five FGs were conducted. The semi-structured interviews, which lasted for an average of 1 h and 15 min, included four interviews with policymakers who were involved with the development or execution of mental health policy either at the state or the national level and six interviews with mental health care professionals, including a psychiatrist, psychologist, counsellors and founders of rehabilitation homes and de-addiction centres. Each FG consisted of six to eight participants and lasted for about 2 h. Four FGs were conducted among the NGO staff members working with various NGOs across Tamil Nadu, and one FG was conducted with the clients of the NGOs. For the client FG, individuals with compromised autonomy (those who are unable to make an informed decision) were excluded.

Through our FGs and interviews, we identified three key areas that influenced the care-seeking behaviour of individuals with mental illness in Tamil Nadu: views on what causes and/or constitutes mental illness, stigma and discrimination associated with mental illness and broader factors influencing decision-making.

### Views on what causes and/or constitutes mental illness

Mental illness was generally associated with non-physiological causes which resulted in little awareness about mental illness:

‘Mental illness may be seen as a negative character flaw that could be caused by God, black magic “Suniyam vachitanga”, supernatural causes “Pai puduchiduchu”, or evil spirits/planetary influence such as “Kiraga Kolaru” and thus must be treated through prayer and other religious means.’ (Psychiatrist, Villivakkam, Tamil Nadu, India)

Given the strong links to religious and/or supernatural causes, individuals with mental illnesses often seek solutions through temples, mosques or through consultation with astrologists.

Furthermore, even for defined problems like alcoholism, there is a lack of general recognition that they constitute a mental illness and defined treatments are not actively sought:

‘Alcoholism is given even less attention as a mental health problem because it is considered the fault of the alcoholic rather than being attributed to genetic or environmental factors. Alcoholics assume they can end their addiction on their own without needing to go to a de-addiction centre.’ (Counsellor at a de-addiction centre, Alwarthirunagar, Tamil Nadu, India)

### Stigma and discrimination associated with mental illness

There is strong stigma and discrimination against the labels of mental illness at multiple levels, including the individual, family and community and society, more generally.

‘Families and the public are likely to see the mentally ill as a burden and offer them little support. In the words of one interviewee, “in my experience there is lot of stigma associated with mental illness and whether it is personal relations, outside your family or it is professional relations, they sideline you if they know that you are mentally ill and even after recovery there is lot of issues in people letting in to the circle again.”’ (NGO client with lived experience of mental illness, Chennai, Tamil Nadu, India)

There are strong social, cultural and economic reasons for the stigma associated with mental illness:

‘Some families worry that if a son or daughter is diagnosed as mentally ill, they will have a more difficult time becoming married. Consequently, they either discourage them from seeking treatment or hide the diagnosis until after the married couple has a child and is more likely to stay married, otherwise the marriage might be dropped if someone knows any of the members in the family have mental problems. They have a fear that if someone in the family has a mental problem the other person in the family might also get affected (contagious).’ (NGO staff member, Chennai, Tamil Nadu, India)

‘Patients who reveal that they are mentally will also face difficulties acquiring employment.’ (NGO client with lived experience of mental illness, Chennai, Tamil Nadu, India)

These different types of stigma manifest in a variety of different forms of discrimination ranging from mild forms like family members just ignoring the individual with the mental illness to more extreme forms of discrimination:

‘In village if a patient became violent people will beat him hard & tie him in a cow shed at night & next day they may take him to hospital in a bus/cart. Till that time no one bothers whether the patient is dead or alive.’ (NGO staff member, Chennai, Tamil Nadu, India)

The stigma and discrimination associated with mental illness creates a fear in individuals and families that significantly affects care-seeking. Indeed, treatment is often times actively avoided because it would lead to a label of ‘mentally ill’ being associated with the individual and the need for the family to acknowledge and accept that a member of their family is mentally ill.

‘Patients who receive treatment but then are no longer accepted by their families. Ultimately, patients delay or avoid mental health treatment to avoid the label of being mentally ill.’ (NGO staff member, Chennai, Tamil Nadu, India)

### Broader factors influencing decision-making

Multiple factors, including stigma, knowledge and expectations, lack of access to practical treatment options and cost, strongly influence the decisions about seeking care. These factors are not mutually exclusive—they interact with each other to create a general perception and attitude about mental illness and care-seeking.

#### Stigma-driven decisions

Stigma associated with mental illness drives individuals and families to avoid acknowledging their illness and, in many instances, to actively seek non-stigmatized labels based on religion or black magic. These stigma-driven decisions negatively influence the care-seeking behaviour and either end up delaying care or resulting in care not being sought at all.

‘With alternative treatments at religious institutions and/or astrologists, patients could spend ample time and money while prolonging delays in seeking medical treatment and reducing their chances of recovery. By the time they come to us it will be 2 to 3 years and their illness is fully bloomed.’ (NGO staff member, Tirupattur, Tamil Nadu, India)

‘Due to the stigma associated with the label of being mentally ill, many sufferers are discouraged from seeking medical care either by their families or because they themselves do not want to. Many people do not know that they are suffering from a mental illness and only when the illness reaches sufficient severity do they or the people around them realize medical treatment is necessary.’ (Psychiatrist, Peelamedu, Tamil Nadu, India)

#### Lack of access to practical treatment options

As with the other areas of India, support options, including treatment, is not always available:

‘Another barrier to accessing mental health care is the lack of providers who are able to treat and manage mental illness.’ (Psychiatrist, Peelamedu, Tamil Nadu, India)

Where support options are available, they may not actually be practical for the individual to access.

‘There are also other factors that prevent the mentally ill from seeking care such as the distance to the nearest facility and cost of transportation. There are no proper ambulance services specifically designed for transporting the mentally ill to mental health treatment facilities. For example, there is no bus transportation from Thiruvalangadu to Thiruvallur district hospital, making it difficult for patients in that area to acquire medical care. Mentally ill patients may also be denied public transport lest they commit violent acts while on board.’ (Policymaker, Chennai, Tamil Nadu, India)

#### Knowledge and expectations

Even where treatment options did exist and there is acceptance that an individual may have a mental illness, there is sometimes a lack of knowledge: that their condition is treatable, that support (treatments, employment support, legal aid, etc.) was available to them and about realistic expectations of what the treatment options can deliver.

‘In both rural areas and cities, there seems to be little awareness about the fact that such disorders are treatable. The public also needs to understand that mental illness takes time to cure and cannot be resolved immediately like other disorders.’ (NGO staff member, Chennai, Tamil Nadu, India)

Where treatment options were available, there was sometimes a general lack of awareness among patients about where to go and whom to approach for support for the various difficulties the individual may face stemming from his/her mental illness.

#### Cost

An important consideration about care-seeking decisions is the cost and the associated opportunity cost which is associated with care—the burden of treatment.

‘Another reason patients avoid seeking treatment for mental illness could be because they are managing other day-to-day problems associated with low income. As mentioned by one interviewee, “the other thing is that already there exist physical problems and problems with the basic needs like water, food, toilets, housing, etc. After overcoming these problems only, the thought of mental health care needs is arising in the mind. When the basic needs are not fulfilled up to the mark, the priority of mental health care needs is settling behind.”’ (Psychiatrist, Villivakkam, Tamil Nadu, India)

The opportunity cost of seeking and receiving mental health care treatment where no practical treatment options are available is a serious concern. Long travel times are a key barrier which drives individuals to avoid seeking care in the first place or to quit treatment after it has started. For example, transport can sometimes take a full day and require substantial resource—to travel from the rural village of Thiruvalangadu to a hospital in Thiruvallur takes more than three buses and over 20 km of transport to and from the bus stands.

If individuals and families are struggling to provide food for their families, they are not likely to invest sufficient resources (time and money) to take care of their mental health.

## Discussion

### Main finding of this study

The key findings of our qualitative study were the identification of three key areas that affect the care-seeking behaviour of individuals with mental illness in Tamil Nadu: views on what causes and/or constitutes mental illness, stigma and discrimination associated with mental illness and broader factors influencing decision-making.

### What is already known on this topic

It is already well known that many individuals with mental illness in Tamil Nadu, and India more generally, do not access mental health care for a variety of reasons, including stigma, discrimination and lack of services.^10–12^^,^^14–19^

### What this study adds

Our study is unique in that multiple stakeholders across the mental health care pathway were engaged to better understand the factors affecting the care-seeking behaviour. Through this approach, we identified specific contextual factors that influence care-seeking behaviour, such as the religious and supernatural interpretation of mental illness by individuals and families; the social, economic and cultural manifestations of stigma and the variety of factors that affect decision-making, including the role of stigma, knowledge and expectations, access to practical treatment options and cost. Overlaid onto these contextual factors, there are three design elements that could be used to develop and implement approaches that can help to address some of the issues that influence care-seeking behaviour in India.

First are the bi-directional links between mental illness and social determinants, such as access to transport, employment and education, which are important considerations for public health in India more generally, particularly when addressing issues related to inequality. Second are the intersectionalities between these different factors in influencing care-seeking behaviour. It is unlikely that the care-seeking behaviour will be addressed by single factors and, to be efficient and effective, interventions will ideally need to address several contextual factors at once. Finally, there are the socio-cultural aspects and tremendous diversity present in India as exemplified by its large number of traditions, cultures, languages, norms, etc. Though the contextual factors identified in this manuscript are likely to be present across India and for different demographic groups, the huge diversity in India means that they will manifest themselves in different ways, which means that interventions will need to be designed to take this diversity into account in order to be effective. Accounting for these design elements when developing interventions to address the contextual factors identified in this manuscript could yield a ‘tool box’ of interventions that could be used in different ways by different stakeholders and for different subgroups of the population to increase the chances of being able to use the right intervention, at the right time, for the right patient.

We hope that the insights of our work can inform operational policies and tactical approaches that can be developed and implemented to reduce the variation in care-seeking behaviour, manifesting in less unmet need and greater equity of access and outcomes for individuals suffering from mental illness in India.

### Limitations of this study

There are three key limitations for our study. Firstly, we were only able to look at one state in India, Tamil Nadu. Secondly, given the ethical considerations, we could only engage individuals who were already part of the patient care pathway (specifically, they were accessing services through an NGO). Finally, we had a limited sample of individuals with mental illness to draw from for our FGs. Despite these limitations, we feel that the methods and insights contributed by this manuscript are valuable more generally for India because they provide contextual information that can help policymakers and practitioners to design and implement approaches to address the key factors which influence the care-seeking behaviour for individuals with mental illness in Tamil Nadu, some of which will likely be applicable in other states of India as well and in other resource-poor settings.

#### Funding

This work is supported by Global Health Policy Programme, Green Templeton College, University of Oxford.

#### Supplement Funding

Oxford-India Sustainable Centre, Somerville College, University of Oxford.

## Supplementary Material

Factors_mental_illness_in_India_Supplementary_Material_1_fdab131

## Notes

**Anant Jani**, Research Fellow

**Sindhu Ravishankar**, Research Assistant

**Naresh Kumar**, Physician

**J. Vimitha**, Research Assistant

**Soleil Shah**, Medical Student

**Anees Pari**, Public Health Consultant

**C. Ramasubramaniam**, State Nodal Officer

**Premila Webster**, Professor
