# Supplementary material for: Factors influencing care-seeking behaviour for mental illness in India: a situational analysis in Tamil Nadu
Source: J Public Health (Oxf). 2021 Oct 8;43(Suppl 2):ii10–6. doi: 10.1093/pubmed/fdab131 (PMC8832222; doi:10.1093/pubmed/fdab131)
Supplement: Factors_mental_illness_in_India_Supplementary_Material_1_fdab131 [file factors_mental_illness_in_india_supplementary_material_1_fdab131.docx]

**Supplementary Material 1 – Interview and Focus Group Guides**

Focus Group Guide

1. Can you all briefly introduce yourself and tell me how you got involved with _______________(NGO name).
2. How do you define mental illness?
3. How important do you think mental health is compared to other illnesses? Why is that? (Prompt: For example, HIV/AIDS, TB and malaria).
4. How does mental health services compare to other types of health care? (Probe: treatment of patients, availability and accessibility of services)
5. How does the general public in this country view mental illness? (Probe: Is there a need for change in public opinion? How can this be achieved?)
6. What key initiatives are needed to address stigma and discrimination toward people with mental health problems? (Prompt: *E.g., anti-stigma campaigns, support for user advocacy/organisation, inclusion in government activities, etc)*
7. What are some of the reasons that people with mental illness do not get the help they need? (probe: policy issues, funding, stigma/community perceptions)
8. What gaps need to be addressed so that patients get the help they need? Where are these gaps. Probe: at the policy level, clinic level, society/family level?
9. Who should be accountable for making sure people living with mental illness are getting adequate support? (Probe: clinics, NGOs, national government, State level government)
10. Describe the extent of availability of mental health services? How easily are people able to access these services? What are some of the reasons for limited availability or lack of access?
11. Can you comment specifically on the availability and access to medication? Who or what agencies offer services to people with mental illness (probe: in the clinic, NGOs, government programs)
12. What is the best source of support for people living with mental illness? (Probe: clinic, NGOs, government programs, family/community support)
13. What is the process around getting a person assessed for mental illness and admitted into a clinic or related facility?
14. What is the process of treating a patient? Probe: how long can a patient stay in the clinic? How long can they be retained on services? What kinds of staff interact with the patient (ie doctors, nurses, counsellors)
15. What are some programs or policies that specifically address women?
16. What are programs or policies that specifically address children and adolescents?
17. What opportunities do people living with mental illness have in influencing state or national level policy?
18. What can you tell me about any national or state level mental health bills or policies? (Probe: What do you know about the national mental health bill?)
19. Do you have any questions of additional comments about mental health in your state that you would like to share?

Interview Guide- Health Care Workers

1. Can you briefly introduce yourself; tell me about your background and your interest in mental health?

***A. I would like to ask you some general background questions***

1. How do you define mental illness?
2. Can you please describe the training you received to work as a mental health provider?
3. How much of the population in your state would you say is affected by mental illness?
4. How important is mental health compared to other illnesses? Why is that? (Prompt: For example, HIV/AIDS, TB and malaria).
5. How does the general public in this country view mental illness? Is there a need for change in public opinion? How can this be achieved?
6. How does mental health provision in your state compare with other health service provision? (Prompt: *For example – differences in funding patterns; differences in media coverage; perceived mutual links with poverty)*
7. How much do you think the State spends on mental health provision? How much more or less do you think should be spent? Why?
8. Who are the other stakeholders/actors in mental health and what can they do to improve the state of mental health?
9. Are mental health services integrated into any other facilities? (probe: schools, alcohol/drug rehabilitation programs, antenatal clinics, other clinics)

***B. Now I would like to ask some questions about mental health policy***

1. What do you know about mental health policies and laws at the state or national level?
2. What influence does the state have at the clinical level with regards to mental health delivery? What are some policies that facilitate identifying people with mental illness and providing appropriate care? What are some policy issues that are barriers for identifying people with mental illness and providing appropriate care?
3. What kinds of platforms or opportunities do health care workers/clinics have to influence national or state level mental health policy? What about people with mental illness?
4. What key initiatives are needed to address stigma and discrimination toward people with mental health problems? (Prompt: *E.g., anti-stigma campaigns, support for user advocacy/organisation, inclusion in government activities, etc)*
5. What are some of the reasons that people with mental illness do not get the help they need? (probe: policy issues, funding, stigma/community perceptions)
6. What gaps need to be addressed so that patients get the help they need? Where are these gaps. Probe: at the policy level, clinic level, society/family level?
7. Who should be accountable for making sure people living with mental illness are getting adequate support? (Probe: clinics, NGOs, national government, State level government)
8. How have the following been addressed, who has addressed them?
   1. Prevention of mental illness
   2. Awareness and stigma reduction
   3. Sensitizing government officials
   4. Implementation of public health programs for care of mental health patients
9. What can you tell me about any national or state level mental health bills or policies? (Probe: What do you know about the national mental health bill?)

***C. I have some questions about mental health provision. Some of the questions are about general service provision, and some are questions about the services your clinic provides.***

1. At what level of care (primary, secondary, etc.) is most healthcare delivered, and what would be optimal?
2. Describe the extent of availability of mental health services? How easily are people able to access these services? What are some of the reasons for limited availability or lack of access?
3. Can you comment specifically on the availability and access to medication? Probe: psychotropic, antipsychotic, antidepressant
4. What are some issues with staffing in clinics in general and at your clinic? Do they have the required skills? If not, where are the gaps? How can these gaps be addressed?
5. What kinds of services or programmes do you offer your patients?
6. How are patients referred to you and what is the process of enrolling a patient?
7. What is the process around getting a person assessed for mental illness and admitted into a clinic or related facility?
8. What is the process of treating a patient? Probe: how long can a patient stay in the clinic? How long can they be retained on services? What kinds of staff interact with the patient (ie doctors, nurses, counsellors)
9. Are there any programs or policies within your clinic that specifically address women?
10. Are there any programs or policies within your clinic that specifically address the needs of children and adolescents?
11. How do your programs or services affect the wider needs of those living in poverty?
12. Where does your clinic get funding? How is this funding allocated? Probe: How does your funding level affect services you provide? If funding is cut, what aspects of service are cut?
13. How do you connect patients with other services they may need? What agencies do you collaborate with?

***D. I just have a few concluding questions***

1. Do you have any additional comments you would like to share about mental health in your state?
2. Can you suggest other individuals we need to talk to?

Interview Guide- Policymakers

1. Can you briefly introduce yourself; tell me about your background and your interest in mental health?

***A. I would like to ask you some general background questions***

1. What are the main social and development priorities in the state? (Prompt: *If health is not mentioned, ask what the position of health is)*
2. Can you explain to me how health services are organised at the state level?
3. What are the key challenges that face the health system in the state?

***B. Now I would like to ask you some questions about mental health***

1. How important is mental health for the government compared to other health conditions? Why is that? (Prompt: For example, HIV/AIDS, TB and malaria).
2. How does mental health provision compare with other health service provision?

(Prompt: *For example – differences in funding patterns; differences in media coverage; perceived mutual links with poverty)*

1. How much do you think the State spends on mental health provision? How much more or less do you think should be spent? Why?
2. How does the state allocate funding for mental health. Where does this funding go? (Prompt: clinics, support to other agencies, medication access)
3. How does the general public in this country view mental illness? Is there a need for change in public opinion? How can this be achieved?
4. What key initiatives are needed to address stigma and discrimination toward people with mental health problems? (Prompt: *E.g., anti-stigma campaigns, support for user advocacy/organisation, inclusion in government activities, etc)*
5. What are some of the reasons that people with mental illness do not get the help they need?
6. What gaps need to be addressed so that patients get the help they need? Where are these gaps. Probe: at the policy level, clinic level, society/family level?

***C. Now I would like to ask you some questions about mental health policies***

1. What are the policies around getting a person assessed for mental illness and admitted into a clinic or related facility?
2. Can you tell me about any policies or programmes in the state outside of health that have an influence on mental health? *For each policy mentioned:* How does that policy affect mental health? (Probe: *For example - education, social welfare, prisons, and women affairs.) Where can these policies be obtained?*
3. What can you tell me about any national or state level mental health bills? (Probe: What can you tell me about the new mental health bill?)
4. (If the interviewee is familiar with the mental health bill) How will the new mental health bill have an impact on mental health policy delivery, etc. in your state?
5. Is there a state mental health policy? *If yes:* Is it different from the national mental health policy? How does it differ from/relate to the national mental health policy? (Prompt: *Where is it set out? (Which documents?) When was it developed?) Request copy of policy*
6. *(If there is a state mental health policy)* How was the policy developed? (Probe: *Stages of policy development, Participatory nature, Use of evidence)*
7. (If there is no state mental health policy) What have been the barriers to developing a state mental health policy?
8. Do you feel the existing mental health policies are adequate? How can they be improved?
9. What sort of input, if any, does the state have in the development of national mental health policy? Have you provided input to the Mental Health Policy document drafted by the National Directorate for Mental Health? (Probe: *Forms of participation, Individuals or state organisations that participated, What stages of development were they involved (policy setting, policy development, or policy implementation)*
10. How well do the mental health policies and laws address the wider needs of people living in poverty? How can the situation be improved?
11. What are some policy issues that specifically affect children and adolescents?
12. What are some policies that affect men and women differently? *(*Prompt*: Explore gender related issues)*
13. Who should be accountable for the state of mental health? Probe: NGO’s, governments, clinics? Why?
14. What kinds of platforms or opportunities do people suffering from mental illness have to influence policies or programmes? Who provides these platforms or opportunities?

***D. Now I would like to ask you about how mental health policies and laws are implemented.***

1. How do national mental health laws and policies take form at the state level? Prompt: To what extent is the national level policies’ fidelity retained at the state level?
2. What process is followed to implement mental health policies in the state? Do you think it is effective?
3. What are the key challenges that face the state health department in implementing mental health policies?
4. How are mental health policies translated into plans and budgets at the state level? Is this effective?(*Probe for examples)*
5. Who are the important organisations or individuals involved in implementing mental health policies in the state? (Prompt: *List individuals and organisations))*
6. What individuals or organisations are not involved in the implementation of mental health laws and policies, but you think should be?

***E. I just have a few more concluding questions for you***

1. Are there any other comments you would like to make about the about mental health policies in your state, at the national level?
2. Do you have any reports or documents that we might find useful for this research, for example, any statements of policy and objectives, annual reports? (Prompt: *Only collect if we do not yet have access to the document)*
3. Can you suggest other individuals who we need to interview?
